# Transducin Duplicates in the Zebrafish Retina and Pineal Complex: Differential Specialisation after the Teleost Tetraploidisation

**DOI:** 10.1371/journal.pone.0121330

**Published:** 2015-03-25

**Authors:** David Lagman, Amalia Callado-Pérez, Ilkin E. Franzén, Dan Larhammar, Xesús M. Abalo

**Affiliations:** Department of Neuroscience, Science for Life Laboratory, Uppsala University, Uppsala, Sweden; University Zürich, SWITZERLAND

## Abstract

Gene duplications provide raw materials that can be selected for functional adaptations by evolutionary mechanisms. We describe here the results of 350 million years of evolution of three functionally related gene families: the alpha, beta and gamma subunits of transducins, the G protein involved in vision. Early vertebrate tetraploidisations resulted in separate transducin heterotrimers: *gnat1/gnb1/gngt1* for rods, and *gnat2/gnb3/gngt2* for cones. The teleost-specific tetraploidisation generated additional duplicates for *gnb1*, *gnb3* and *gngt2*. We report here that the duplicates have undergone several types of subfunctionalisation or neofunctionalisation in the zebrafish. We have found that *gnb1a* and *gnb1b* are co-expressed at different levels in rods; *gnb3a* and *gnb3b* have undergone compartmentalisation restricting *gnb3b* to the dorsal and medial retina, however, *gnb3a* expression was detected only at very low levels in both larvae and adult retina; *gngt2b* expression is restricted to the dorsal and medial retina, whereas *gngt2a* is expressed ventrally. This dorsoventral distinction could be an adaptation to protect the lower part of the retina from intense light damage. The ontogenetic analysis shows earlier onset of expression in the pineal complex than in the retina, in accordance with its earlier maturation. Additionally, *gnb1a* but not *gnb1b* is expressed in the pineal complex, and *gnb3b* and *gngt2b* are transiently expressed in the pineal during ontogeny, thus showing partial temporal subfunctionalisation. These retina-pineal distinctions presumably reflect their distinct functional roles in vision and circadian rhythmicity. In summary, this study describes several functional differences between transducin gene duplicates resulting from the teleost-specific tetraploidisation.

## Introduction

Phototransduction is the process whereby light is converted into electrochemical signals. It predominantly takes place in photoreceptor cells, but also in other non-specialised cell types. There are two main mechanisms of phototransduction in the animal kingdom: the Gαt and Gαq signalling pathways, used by ciliary and rhabdomeric photoreceptor cells, respectively. The present work is focused on the vertebrate-specific ciliary photoreceptors, the rods and cones, and their phototransduction process.

**Fig 1:**
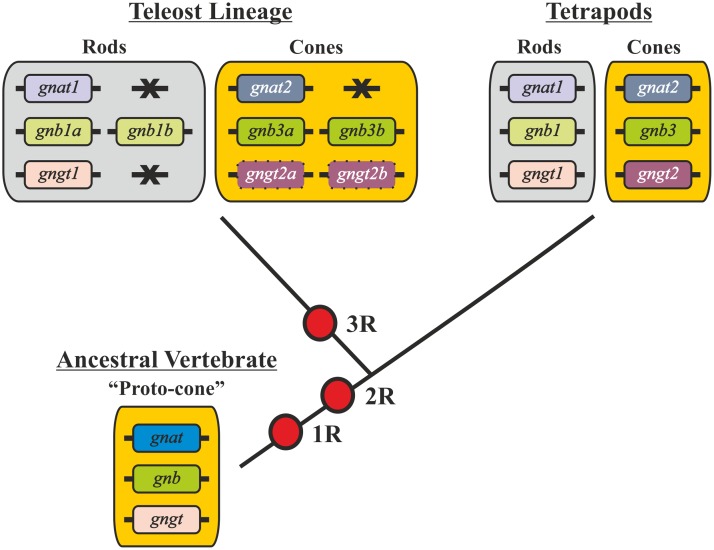
Phylogenetic cladogram summarising the evolution of the transducin subunit genes within vertebrates. The three transducin subunit genes present in the vertebrate ancestor cone-like photoreceptors (“proto-cones”) (*GNAT*, *GNB* and *GNGT*) gave rise to two paralogs for each gene represented in extant tetrapods after 1R and 2R: *GNAT1*-*GNAT2*, *GNB1*-*GNB3* and *GNGT1*-*GNGT2*. This facilitated the appearance of rods. In teleost fishes the 3R tetraploidisation resulted in three pairs of paralogs that have been retained in zebrafish: *gnb1a*/*gnb1b*, *gnb3a*/*gnb3b and gngt2a*/*gngt2b*.

Retinal rods and cones are responsible for scotopic and photopic vision, respectively. Both photoreceptor types have been suggested to have originated from an ancestral cone-like photoreceptor (proto-cone [1]), see Fig. 1), after the two rounds of whole genome duplication, or tetraploidisation, (1R and 2R) that occurred early in vertebrate evolution [2, 3]. After such a duplication event, the duplicated genes (paralogs) may be deleted or retained. The retention of duplicates may result from mutations causing differential functional interactions of the gene products or differential expression of the two paralogs [4]. Therefore, there are two possible outcomes; subfunctionalisation, whereby the functions and/or expression of the ancestral gene are partitioned between the duplicates, or neofunctionalisation, whereby one or both of the copies gain novel functions [5, 6].

Light perception through ciliary photoreceptors occurs mainly in the retina, however, it is also found in the pineal complex of non-mammalian vertebrates, where it does not have an image-forming role. It is not clear whether the pineal complex represents a primitive stage in eye evolution or if it has evolved independently of the vertebrate eye [7]. Both rod-like and cone-like photoreceptors have been described in the pineal gland of non-mammalian vertebrates [8–11].

Both the retina and the pineal complex contain light-sensitive cell types, however, our knowledge about the phototransduction cascade components of these cells is incomplete for the eye, as well as scarce and ambiguous for the pineal complex. Many of the genes encoding the components of the vertebrate phototransduction cascade are members of gene families that expanded in 1R and 2R [12–15]. This suggests that different rod- and cone-specific phototransduction cascade proteins evolved from a common ancestral cascade through the diverging specialisation of paralogs. The present work investigates the functional specialisation of the G proteins involved in the phototransduction cascade: the transducins. To this end, we have analysed and compared the expression of the genes encoding the transducin subunits, both within the retina and between the retina and the pineal complex.

Transducins are heterotrimeric proteins that consist of α, β and γ subunits, whose genes are named *GNAT*, *GNB* and *GNGT* for guanine nucleotide-binding proteins alpha, beta and gamma, respectively. Transducins are activated after the conformational change of the opsin upon excitation by light, which initiates an intracellular relay of biochemical changes that leads to the hyperpolarisation of the photoreceptor cells. Our research group recently published an evolutionary study of the three transducin subunit gene families [14]. By combining sequence-based phylogenies with chromosomal location data we concluded that all three transducin subunit gene families expanded in 1R and 2R, and that the beta and gamma subunit families gained additional members in the third round of whole genome duplication (3R) that occurred early in teleost fish evolution [16].

Expression pattern analyses provide clues about the specialisations of paralogous genes. According to the general paradigm, rods and cones express distinct variants of the three transducin subunits: rods express *GNAT1*, *GNB1* and *GNGT1*, whereas cones express *GNAT2*, *GNB3* and *GNGT2* [17, 18] (see Fig. 1), and several studies in different species have used transducin subunits as photoreceptor-specific markers [19, 20]. However, in teleost fishes additional paralogs are now known to exist. In particular, the zebrafish has retained duplicated 3R paralogs of the *gnb1*, *gnb3* and *gngt2* genes: *gnb1a/gnb1b*, *gnb3a/gnb3b* and *gngt2a/gngt2b* ([14], see summary in Fig. 1).

Among the teleosts, the zebrafish has become the prime model for genetic, developmental and functional studies of the visual system [21–24], as well as for evolutionary studies [14, 15, 25]. Additionally, there are some studies relating the transducin gene expression to the early development of the visual system in zebrafish [21, 26–29], and/or knocking out some of these genes (*gnat1* [30], *gnat2* [31], *gnb1a*, *gnb1b* [30], *gngt1*, *gngt2a*, *gngt2b* [32]). However, a complete expression pattern analysis of all the transducin gene duplicates through the life span is lacking.

The present study was designed to provide an evolutionary perspective on the specialisation of transducin paralogs, focusing on rods versus cones in the retina and possible specialisations in the pineal complex. The expression patterns of the transducin genes in zebrafish were analysed during the ontogeny as well as in the adult stage, giving special attention to the gene duplicates retained after 3R: *gnb1a/gnb1b*, *gnb3a/gnb3b* and *gngt2a/gngt2b*.

## Materials and Methods

This study was carried out in strict accordance with the recommendations of the Federation of Laboratory Animal Science Associations. The project was approved by the Uppsala Ethical Committee on Animal Experiments (Uppsala djurförsöksetiska nämnd), permit numbers: C33/10, C294/12 and C315/12. Prior to any procedure, all animals were anaesthetised with benzocaine (0.5 ml/L).

### Zebrafish embryos, larvae and adults

The embryos and larvae used in this study were AB wild type and albino strains. All embryos and larvae were collected and prepared for gene expression analyses as described previously [33]. The adult animals were 6 months to 1 year old AB zebrafish (n = 58), purchased from the Science for Life Laboratory Zebrafish Technology Platform (Uppsala University), or cone-specific transgenic zebrafish, Tg(*gnat2*:EGFP) [34], provided by Dr. B. Kennedy (University College Dublin) (n = 6).

### Expression pattern analyses

Expression pattern analyses were performed by *in situ* hybridisation (ISH) on sections of adult zebrafish heads or whole-mount (WISH) embryos and larvae, using riboprobes specific to all zebrafish transducin subunit mRNAs. The following sequences were used as templates for primer design: *gnat1* (ENSDART00000064896), *gnat2* (ENSDART00000062363), *gnb1a* (BC071277.1/NM_212609.1), *gnb1b* (ENSDART00000084989), *gnb3a* (ENSDART00000012673), *gnb3b* (ENSDART00000005547), *gngt1* (ENSDART00000051950), *gngt2a* (ENSDART00000024136) and *gngt2b* (ENSDART00000122684). All sequences were obtained from the Ensembl 58–59 database.

Standard cloning procedures were applied to synthesise the riboprobes. In summary, specific primers against the 3´ untranslated region (3´UTR) of each gene were used to amplify a 202–550bp sequence by PCR. The obtained amplicon was inserted into a pCRII-TOPO vector and cloned in one shot chemically competent TOP 10 *E*. *coli*. The primer pairs for each transducin subunit gene are shown in Table 1. The use of the 3´UTRs ensures higher specificity, as they hold higher nucleotide variability than the coding regions. Subsequently, all riboprobes (sense and antisense) were synthesised by either T7 or SP6 RNA polymerases using a DIG RNA Labelling Kit (Roche: cat. no. 11175025910), labelling them either with digoxigenin (DIG) or fluorescein (FITC).

**Table 1:** Primer sequences used to amplify the transducin genes.

	Gene	FW primers: 5´-3´	RW primers: 5´-3´	Product
**1**	*gnat1*	5´-TGGCTGAATCAACAAAAC-3´	5´-TCATCCACCTCACATAGACA-3´	524 bp
**2**	*gnat2*	5´-GCCCCATCCCCACCTAA-3´	5´-ATTGCGATCTGATTTCCCACTA-3´	472 bp
**3**	*gnb1a*	5´-AGCCGTAAGACCGCATCCGGA-3´	5´-AGCGTCGCGAAACTCGATGGA-3´	550 bp
**4**	*gnb1b*	5´-GTGTGACCCTGTAAGAGAAAAC-3´	5´-TCACAGGAGGGCGCATAAACATT-3´	432 bp
**5**	*gnb3a*	5´-TCAAAGAAATCACGCAATAACAGA-3´	5´-GGCCCGAATAAGCAGAAGAA-3´	470 bp
**6**	*gnb3b*	5´-CTCCGGAAGACTGGCTGTT-3´	5´-CTGTCTGGCATGTAAAAGT-3´	466 bp
**7**	*gngt1*	5´-GACAGAAAATCCCCCAACAT-3´	5´-TGAACAGCTAAATTACTCCACCAT-3´	400 bp
**8**	*gngt2a*	5´-AGCCTGTCTCTAAAACTG-3´	5´-GTCTTCATGTACTAAAACTAA-3´	214 bp
**9**	*gngt2b*	5´-CAGCAACAGCCCCAGAAATCATTGC-3´	5´-AAAGCATTTCTAGGACCGGCAAACT-3´	202 bp
**10**	*gnat2*	5´-TCGCCATCTGCACAGGAGGAT-3´	5´-GCTCTGGAGGCATGGTGCCC-3´	79 bp
**11**	*gnb1a*	5´-AGATCAGAGATGCGCGGAAAG-3´	5´-CAAGTGTCCCCTCAGTGTCC-3´	116 bp
**12**	*gnb1b*	5´-ACTATCACAGATCACAGCCAACA-3´	5´-GTGCATGGCGTAGATTTTAGC-3´	100 bp
**13**	*gnb3a*	5´-ACGCCATTGGGTTTTTCCCCA-3´	5´-GGACGTCACGCCGCACATGA-3´	137 bp
**14**	*gnb3b*	5´-CACAGATTGAGGCGGCTCGCA-3´	5´-AGTTGGACCCGAGGGGCTGG-3´	88 bp
**15**	*bactin I*	5´-GGCACGAGAGATCTTCACTCCCC-3´	5´-CCATGCCAACCATCACTCCCTGA-3´	195 bp
**16**	*tuba I*	5´-CGGAGCTGGAAAACACGTCCCC-3´	5´-TGGTCAGACAGTTTGCGAACCCTA-3´	216 bp

Primer pair sequences used to amplify all zebrafish-specific transducin genes. Primer pairs 1–9 were used to synthesise the antisense and sense riboprobes used in ISH experiments, 10–14 were used to analyse the expression level by RT-qPCR, and 15–16 were used to amplify beta actin I (*bactin1*) and alpha tubulin 1 (*tubaI*) as housekeeping genes.

To prepare the sections for ISH, adult zebrafish heads were dissected immediately after anaesthesia, fixed by immersion in 4% paraformaldehyde diluted in phosphate buffer (0.1M, pH 7.4) for 6 to 8 hours, and washed in phosphate buffer saline (0.1M, pH 7.4) overnight. The tissues were cryoprotected by immersion in 30% sucrose diluted in phosphate buffer and sectioned in a cryostat (Microm Cryo-Star HM 560). Transversal or sagittal sections of the whole head (12–20 μm thick) were obtained and stuck to positive-charged slides.

ISH (n = 20) and WISH (n = 4) were performed according to Hauptmann and Gerster, 2000 [33], with minor adaptations in the case of ISH. The final staining reaction was carried out using different substrates for the AP enzyme bounded to the Fab fragments: NBT/BCIP or Fast Red tablets (Roche: cat. no. 11681451001 and 11496549001, respectively). Double ISH according to Hauptmann, 2001 [35] was also performed in specific cases.

Specific expression was tested in the eye for each gene by RT-PCR using eye cDNA (see description below). To test the specificity of the antisense probes, sense probes were synthesised and incubated in parallel with the antisense probes, obtaining no staining for any of them. In addition, non-labelled antisense probes were used in a competitive reaction with the labelled antisense probes at different concentrations.

**Fig 2:**
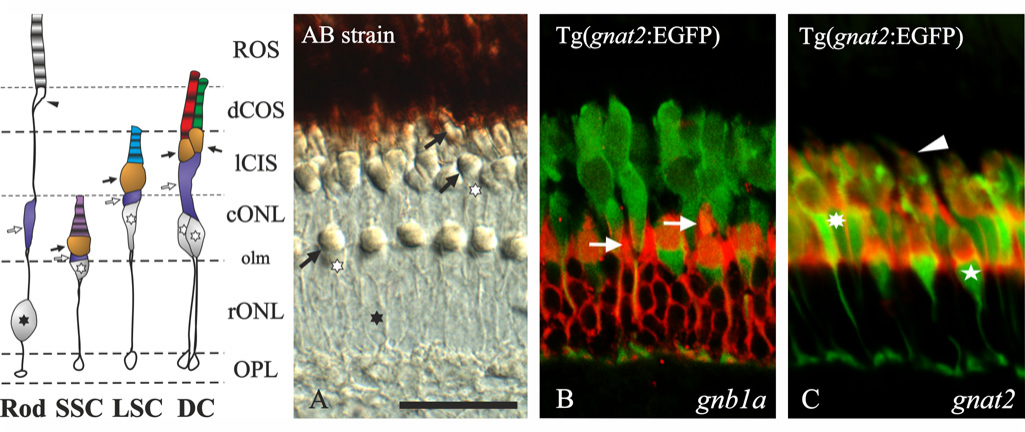
Adult zebrafish outer retina; structure and probe specificity. The schematic diagram of the outer retina to the left is based on Raymond and Barthel, 2004 [36] and a Nomarski contrast photomicrograph from a radial section of an adult AB zebrafish retina (**A**). All photoreceptors have a terminal in the outer plexiform layer (OPL). The outer nuclear layer (ONL) is subdivided by the outer limiting membrane (olm) into a sublayer where the rods have their nuclei (black asterisk: rONL) and a more external sublayer where the cones have their nuclei (empty asterisks: cONL). Note that the short single cones (SSC) have their nuclei in the rONL while their oil droplets (black arrows) and outer segments (OS) are in the cONL. The rods’ myoids (empty arrows) are embedded in the cONL. The long cone inner segment sublayer (lCIS) includes the oil droplets and myoids of the double cones (DC) and the long single cones (LSC) and part of the LSC OS. The outermost part of the retina (top) is covered by the pigmentary epithelium where the DC OS (dCOS) and the rods’ ellipsoid (arrowhead) and outer segments (ROS) are embedded. **B** and **C** are confocal photomicrographs of *in situ* hybridisation experiments performed using Tg(*gnat2*:EGFP) zebrafish, which expresses EGFP in all cones. Specific Fast Red staining in rods for *gnb1a* (**B**) and cones for *gnat2* (**C**) can be observed. Arrows in **B** point to the rod´s myoids that in some cases could be mistaken as SSC. In **C**, the arrowhead points to a DC, the asterisk to a LSC and the star to a SSC. Scale bar in **A** represents 25 μm.

To confirm the cell type assignment for each gene, ISH experiments (n = 6) were performed on Tg(*gnat2*:EGFP) zebrafish (Figs. 2B and C). The cone-specific EGFP fluorescence was enhanced by incubation with a mouse anti-GFP antibody (1:400, Invitrogen: cat. no. A-11120) and a secondary donkey anti-mouse coupled Alexa 488 (1:1000, Invitrogen: cat. no. A-21202). In order to prevent the EGFP decay, the hybridisation time was reduced from overnight to 6 hours. Antibodies against GNAT1, GNB1 and opsins were also used as markers but not included in the study.

### Microscopy and photography

The general anatomy of the zebrafish retina has been described previously in detail [37], and it is known to follow the general organisation pattern common to vertebrates [38]. Our results were analysed based on overall cell morphology, topological location of the nuclei and oil droplets of the different photoreceptor cell types, as well as cone-specific EGFP fluorescence of the Tg(*gnat2*:EGFP) zebrafish (Fig. 2). We have used the nomenclature proposed by Raymond and Barthel, 2004 [36] for the different cone types present in the zebrafish retina: double cones (DC, middle and long wavelength), long single cones (LSC, short wavelength) and short single cones (SSC, ultraviolet).

Bright-field, fluorescence and Nomarski contrast photomicrographs, as well as combinations, were taken for the ISH experiments on slides, using a Zeiss Axioplan 2 microscope equipped with a Zeiss AxioCam camera. In addition, an inverted LSM510 Zeiss confocal microscope was used for detailed pictures. The images of WISH embryos and larvae were acquired using a stereomicroscope Nikon SMZ1500 with a Nikon DS-Vi1 camera All images were processed and the figures merged using CorelDRAW Graphics Suite X6.

### Expression level analyses

Gene expression levels were analysed by reverse transcriptase quantitative PCR (RT-qPCR) for *gnat2*, *gnb1a*, *gnb1b*, *gnb3a* and *gnb3b*, using β-actin I (*bactin1*) and α-tubulin 1 (*tubaI*) as internal control (housekeeping) genes. Adult animals (n = 30) were kept in a 14–10 light-dark cycle and sacrificed at different Zeitgeber (ZT) time points during 24h (ZT0, ZT4, ZT8, ZT12, ZT16 and ZT20). This enables the study of rhythmic oscillations on gene expression without any endogenous interference derived from a dark or light adaptation processes. Total mRNA was extracted from adult eyes (n = 5 per time point) or from whole 1 day post-fertilisation (dpf), 2 dpf and 3 dpf embryos (n = 30) with an RNeasy Mini Kit (Qiagen: cat. no. 74104) precipitated for higher purity, treated with DNase I (Fermentas, cat. no. EN0525) and used for downstream cDNA synthesis in RT and NRT reactions using M-MLV RT Reverse Transcriptase (Invitrogen: cat. no. 28025–013) according to the manufacturer´s instructions.

RT-qPCRs were carried out in a CFX96 RT-PCR detection system (Bio-Rad), using IQ SYBR Green Supermix (Bio-Rad: cat. no. 1708880) and specific primers (Table 1). All samples were analysed in triplicates. No template controls (NTCs) were also performed for each primer pair. A reference pool of cDNA and NRT was also used in triplicate for each plate to control inter-plate variation between different runs. After each RT-qPCR run, a melt curve was made to control for primer dimers and the correct amplicon. The results were compared to the expression of β-actin, as internal housekeeping, using the 2^-ΔCt^ method and analysed using LinRegPCR 2014.6 [39]. The statistical differences were calculated using StatPlus:mac V5 two-way ANOVA and Tukey HSD *post-hoc* test.

## Results

The present study focuses on the specialisations of the zebrafish transducin genes. For all positive cases, the staining was found in the photoreceptors, not in the inner retina, and most of them also in the pineal complex. No specialised expression within photoreceptor types (rods or cones) could be found for any of the transducin gene 3R-generated duplicates. Additionally, stronger intensity was observed in the dorsal retina for the genes with expression in the entire retina, which could be related to the asymmetrical cell distribution and organisation between the dorsal and the ventral retina previously described in zebrafish [40].

### Expression patterns of the transducin genes in the adult zebrafish retina

**Fig 3:**
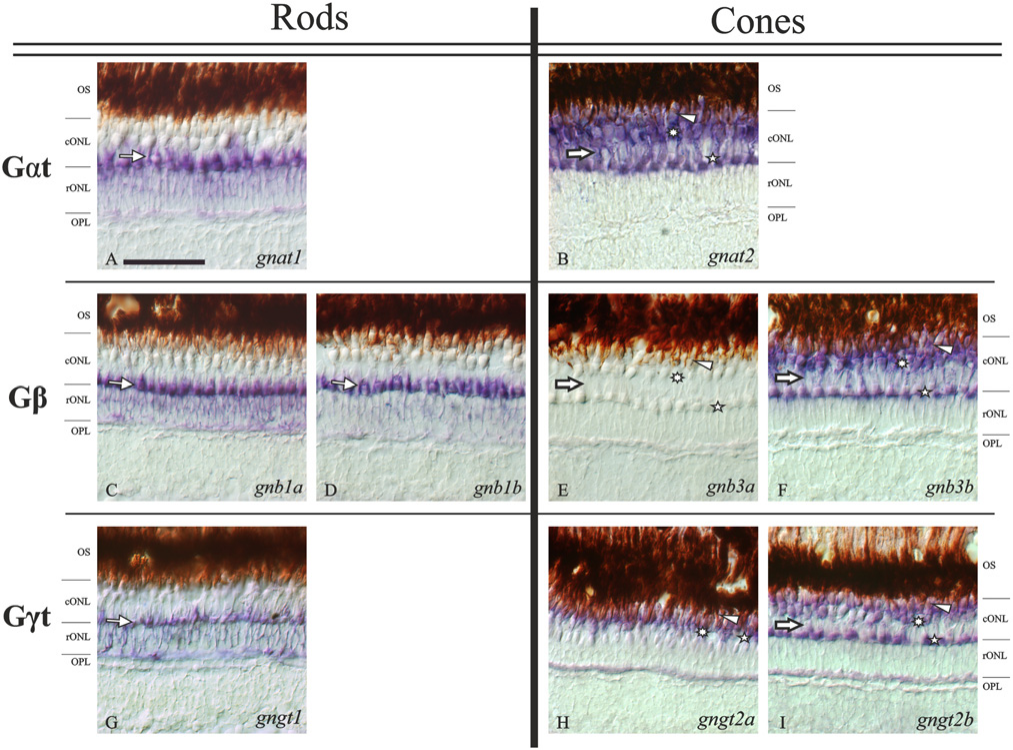
Expression patterns of each transducin subunit gene in the adult zebrafish retina. Nomarski contrast photomicrographs from radial sections of adult zebrafish retina showing the expression of all the transducin mRNAs. Panels **A, C, D** and **G** show the rod-specific expression of *gnat1*, *gnb1a*, *gnb1b* and *gngt1*, respectively. Their expression is observed in the rod nuclei of the rONL, but the strongest staining is observed in their myoids, which are embedded in the cONL sublayer (thin arrows). Panels **B**, **F**, **H** and **I** show the expression of *gnat2*, *gnb3b*, *gngt2a* and *gngt2b*, respectively, in all cones: DC (arrowheads), LSC (asterisks) and SSC (stars). The brown retinal pigment epithelium is shown the uppermost part for each picture. The stratification of the outer retina is evident in all panels except **H**, due to the exclusive ventral expression of *gngt2a*, where the stratification becomes unclear. Panel **E** shows the lack of staining in the adult retina for the *gnb3a* gene. Note that the weaker stained band into the ONLc for the cone-specific transducin subunits (thick arrows) corresponds to the rods’ myoid position (thin arrows in rods photomicrographs). Scale bar in panel **A**: 50 μm. For abbreviations see Fig. 2 legend.

The presence in the zebrafish genome of all the transducin genes: *gnat1*, *gnat2*, *gnb1a*, *gnb1b*, *gnb3a*, *gnb3b*, *gngt1*, *gngt2a* and *gngt2b* was verified by PCR using genomic DNA and the 3´UTR primers designed for each gene (see Table 1). The same primers were also used to investigate the transducin gene expression in the retina by RT-PCR using retina cDNA and confirmed that all transducin genes, except *gnb3a*, are transcribed in the retina. Subsequently, subtype-specific antisense DIG/FITC-labelled riboprobes were used in ISH. Positive staining was observed in the outer retina for all the genes, with the exception of *gnb3a* (Fig. 3). Some of the mRNAs were also detected in the pineal complex (see description below), but not in any other part of the head.

#### 1. The expression of gnat1 and gnat2 genes is restricted to rods and cones, respectively

There are two genes coding for zebrafish transducin alpha subunits: *gnat1* and *gnat2*. These have previously been shown to be rod- and cone-specific, respectively [31]. No duplicate of any of these genes was retained after 3R [14], indicating no further specialisation in teleost fishes. Nevertheless, we analysed their expression patterns to provide a complete picture of the transducin gene expression in the zebrafish retina. *Gnat1* mRNA was restricted to the rod inner segments (Fig. 3A). The staining can be observed in the thin layer of cytoplasm surrounding the nuclei, and more intensely in the rod’s myoid, embedded in the cONL (Fig. 3A). *Gnat2* mRNA was found in the inner segments of all cone types: DC, LSC and SSC (Figs. 2C, 3B).

#### 2. The gnb1 paralogs retained after 3R are co-expressed in adult rods

**Fig 4:**
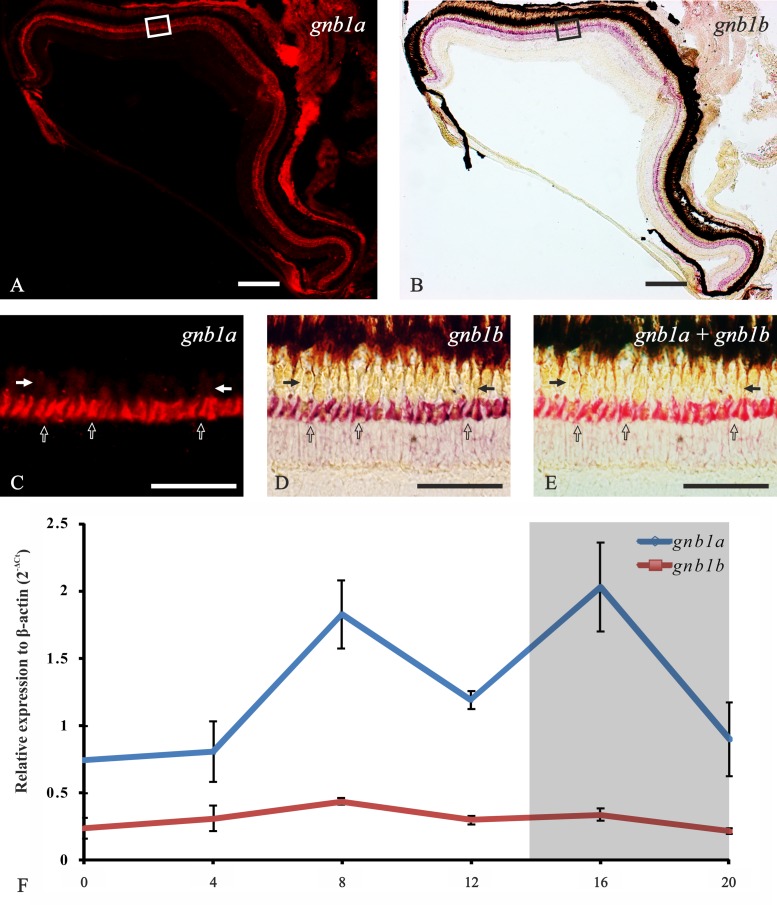
Co-expression of *gnb1a* and *gnb1b* genes in the adult zebrafish retina. Photomicrographs from an adult retina showing combination of *gnb1a* and *gnb1b* antisense riboprobes in a double ISH experiment. The upper row displays radial sections of the entire adult retina (dorsal side to the left), while the middle row shows higher magnification of the area marked within the square in the upper pictures (outermost side at the top). **A** and **C** are fluorescent pictures showing Fast Red staining for *gnb1a*. **D** is a bright-field picture showing mainly NBT/BCIP staining for *gnb1b* in purple but also a minimal red from the Fast Red. **B** and **E** pictures combine bright-field and fluorescence to show the co-expression of both mRNAs in the same rods. Empty arrows in panels **C-E** point at single rods and arrows mark the oil droplets region that gives autofluorescence in panel **C**. Scale bars are 200 μm for panels **A-B** and 50 μm for panels **C-E**. The bottom of the figure displays a graph showing the expression levels of the two genes at different Zeitgeber time points during the day (0 to 20 in X-axis) in relation to β-actin as housekeeping gene (Y-axis), using the 2^-ΔCt^ method (**F**). Note that the expression level of *gnb1a* is significantly higher than *gnb1b* at all time points. Moreover, *gnb1a* expression oscillates, with significantly higher peaks of expression (p<0,05) at ZT8 and ZT16, while *gnb1b* does not oscillate.

**Fig 5:**
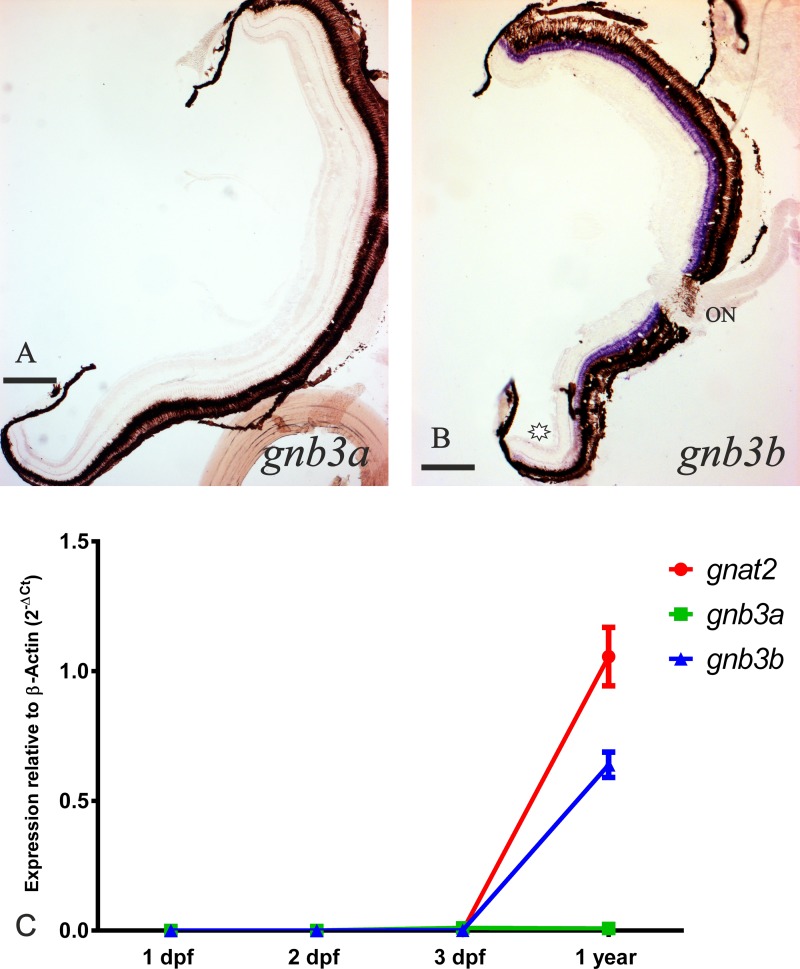
Expression of the *gnb3* paralogs retained in zebrafish after 3R. The upper part of the figure shows two photomicrographs from radial sections of adult zebrafish eyes. The inner side is to the right and the dorsal side to the top. No mRNA could be detected for the *gnb3a* gene (**A**). Expression of the *gnb3b* gene (in purple) was observed in the dorsal and medial retina (**B**), while the ventral retina lacks mRNA (asterisk). The black-brown tissue surrounding the retina corresponds to the pigmentary epithelium. ON; optic nerve. Scale bars: 200 μm. At the bottom of the figure there is a graph comparing the cone-specific *gnat2*, *gnb3a* and *gnb3b* expression levels between 1 dpf, 2 dpf, 3 dpf zebrafish and eyes of 1 year old individuals in relation to the β-actin housekeeping gene using the 2^-ΔCt^ method (**C**). Note the increment in the expression of *gnat2* and *gnb3b* in the adult stage while the similar amount of mRNA for the *gnb3a* gene present in the 3 dpf embryos and an adult eye.

Contrary to the alpha subunits, the transducin beta subunit genes have retained duplicates after 3R ([14], Fig. 1): *gnb1a*, *gnb1b* (Figs. 2B, 3C, D, 4) and *gnb3a*, *gnb3b* (Figs. 3E, 3F, 5A, 5B). Both *gnb1a* and *gnb1b* were found to be expressed in rods (Fig. 3). Interestingly, co-localisation in the same rod photoreceptors was demonstrated by double ISH (Figs. 4C–E). For both genes, the expression is more intense in the dorsal than in the ventral retina (Figs. 4A-B), as is the case for all the transducin mRNAs.

Although ISH is a qualitative method, the analysis of these results suggested different expression levels for the two genes (Figs. 3C, D). This was quantified by RT-qPCR and we found that there is, indeed, a statistically significant higher expression for *gnb1a* at all time-points (p<0,05; Fig. 4F). The expression levels of *gnb1b* are continuous throughout the day, while *gnb1a* shows a slight rhythmic oscillation with two peaks of expression around ZT8 and ZT16 (p<0,05; Fig. 4F).

#### 3. The gnb3 paralogs show different expression patterns after 3R

Gene expression analysis of *gnb3a* and *gnb3b* showed spatial subfunctionalisation (compartmentalisation). Expression of *gnb3b* could be observed in cones of the dorsal and medial retina exclusively (Figs. 3F, 5B). However, expression of *gnb3a* could not be detected in adult retinae (Figs. 3E, 5A). Further RT-qPCR experiments revealed comparable expression levels of *gnb3a* in adult eyes and whole 3 dpf embryos (Fig. 5C), while the expression levels for cone-specific *gnat2* and *gnb3b* showed significant expression increase from 3 dpf embryos to adult eyes.

#### 4. The gngt2 paralogs retained after 3R show compartmentalisation in adult cones

**Fig 6:**
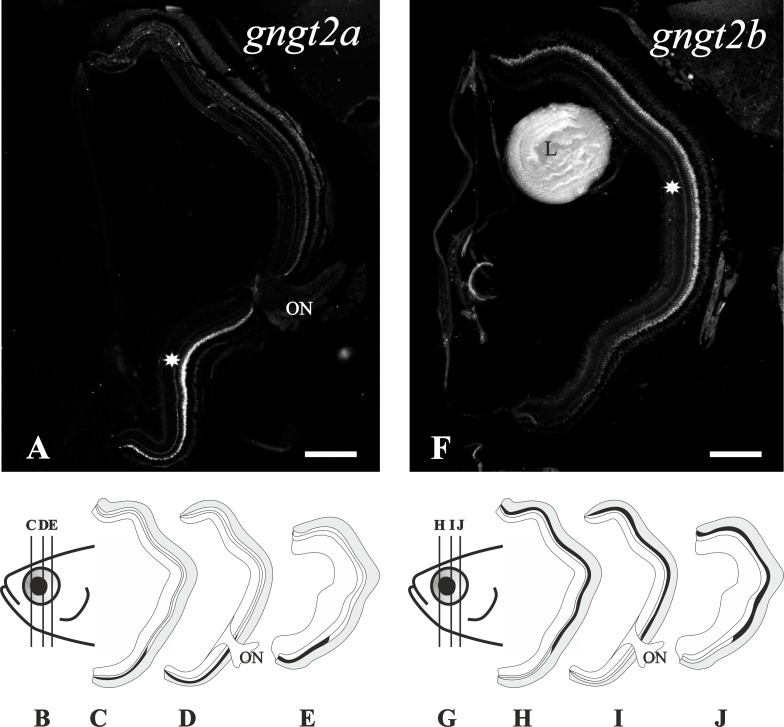
Compartmentalisation of the *gngt2a* and *gngt2b* cone-specific paralogs in the adult retina. The upper part shows two photomicrographs from radial sections of adult zebrafish eyes. The inner side is to the right and the dorsal side to the top. In them, expression patterns (asterisks) can be observed in the ventral retina for the *gngt2a* gene (**A**) and in the dorsal and medial retina for the *gngt2b* gene (**F**). At the bottom are two drawings of a zebrafish adult head in a lateral view (**B**, **G**), pointing out the level where the sections were taken for the schematic drawings in panels **C**, **D** and **E** for *gngt2a*, and panels **H**, **I** and **J** for *gngt2b*. L; lens, ON; optic nerve. Scale bars: 200 μm.

This study shows rod-specific expression of the *gngt1* gene in zebrafish (Fig. 3G), in agreement with results from other vertebrate species [26]. The cone-specific transducin gamma gene *gngt2* was duplicated in 3R and zebrafish, unlike other investigated teleosts, has retained both copies: *gngt2a* and *gngt2b* [14]. Their expression patterns revealed cone-specific restriction (Figs. 3H, I) and compartmentalisation: the *gngt2a* mRNA is only observed in the ventral retina (Figs. 6A–E), while the expression of *gngt2b* is observed in the dorsal and medial retina (Figs. 6F–J). A small overlapping region was observed where both genes are expressed, but always in distinct cone types.

### Expression of five transducin genes was detected in the adult pineal complex

The pineal complex in zebrafish consists of the pineal and parapineal organs, which are located dorsal to the diencephalic roof and connected to it via the pineal stalk. The pineal organ displays a flat T- or Y-shaped structure with a pineal vesicle opened to the third ventricle. It consists of ependymal cells, melanocyte-like cells, fibrous astrocytes and two types of photoreceptor-like cells that represent the morphofunctional unit of the gland [41]. The parapineal organ is an unpaired, left-sided accessory organ that has different names depending on the subclass of animals: parapineal in fish, parietal eye in reptiles and frontal organ in amphibians [42]. In this study we did not use any pineal versus parapineal cell markers, apart from the structural and morphological evidence, so we will refer to the pineal complex unless specified otherwise.

**Fig 7:**
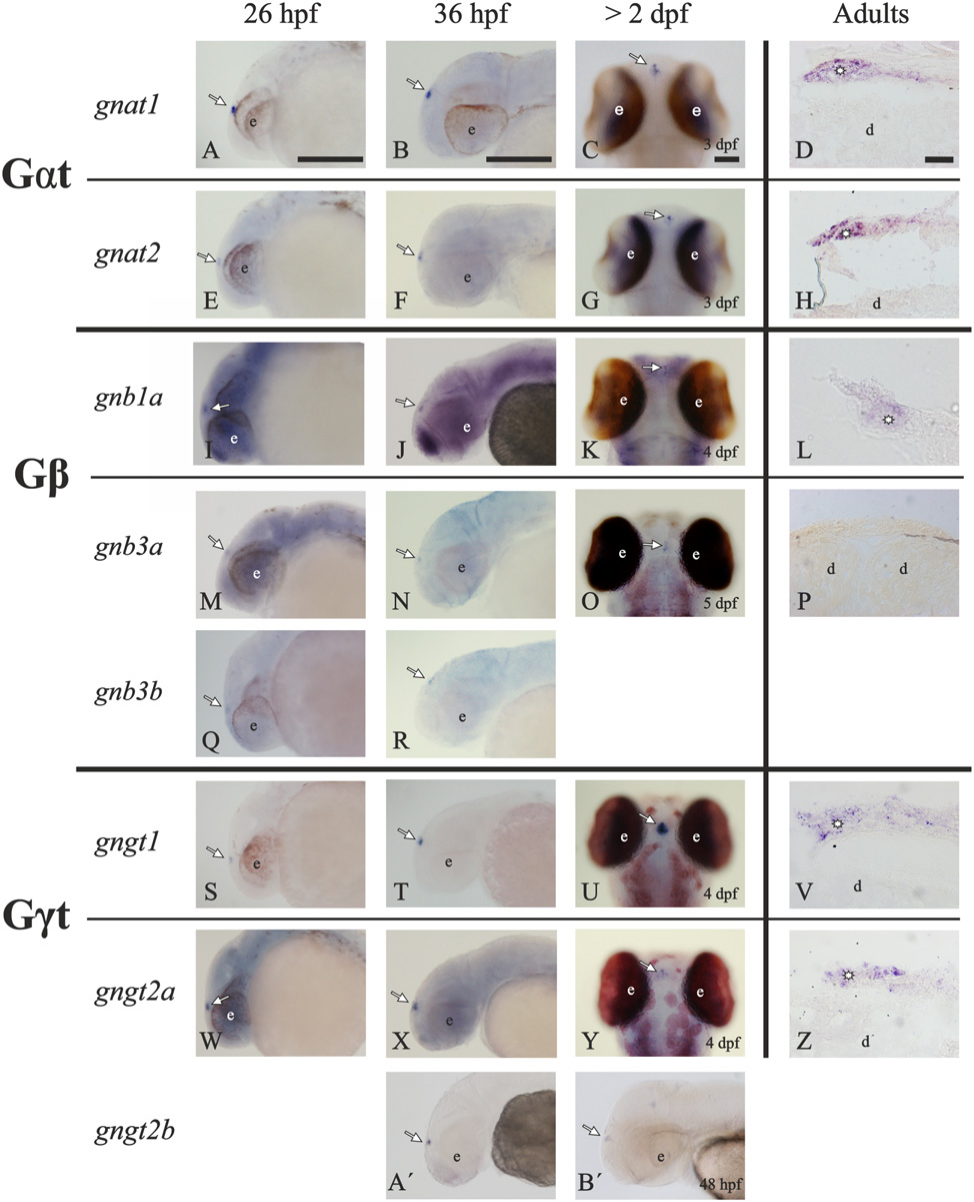
Summary of the transducin subunit gene ontogeny in the zebrafish pineal complex. The photomicrographs show the presence of mRNAs for the transducin genes in the pineal complex of zebrafish. In adults, transversal sections from the dorsal diencephalon show expression of *gnat1* (**D**), *gnat2* (**H**), *gnb1a* (**L**), *gngt1* (**V**) and *gngt2a* (**Z**) in the pineal complex (asterisks) but not in the dorsal diencephalon (d). Observe the complete lack of mRNA for the *gnb3a* gene (**P**). The ontogenetic analysis shows a synchronised onset and development of expression starting at around 26 hpf for *gnat1* (**A-C**), *gnat2* (**E-G**), *gnb1a* (**I-K**), *gnb3a* (**M-O**), *gngt1* (**S-U**) and *gngt2a* (**W-Y**). In addition, there is a transient expression of *gnb3b* (**Q**, **R**) and *gnbt2b* (**A´, B´**) in the pineal complex prior to hatching. Note the background-like expression found in the early stages for *gnb1a* (**I-K**), *gnb3a* (**M**, **N**) and *gngt2a* (**W-Y**). In all cases, arrows point to the location of the pineal complex, and “e” mark the location of the eyes. The photomicrographs in panels **C**, **G**, **K**, **O**, **U** and **Y** show a dorsal view of the larvae, while the others show lateral views. Scale bars: 50 μm in larvae and 30 μm in adults. For abbreviations see Fig. 2 legend.

The present work shows the presence of mRNA for *gnat1* (Fig. 7D), *gnat2* (Fig. 7H), *gnb1a* (Fig. 7L), *gngt1* (Fig. 7V) and *gngt2a* (Fig. 7Z) in the adult zebrafish pineal complex. No expression was detected for *gnb1b*, *gnb3a*, *gnb3b* or *gngt2b*. Expression of *gnb3a* (Fig. 7P) could not be observed in the adult pineal complex; however, it was detected in larval stages (Figs. 7M–O). The *gnb1* expression pattern in the pineal complex provides another example of specialisation, i.e., while the two paralogs are co-expressed in the rods of the retina (Fig. 4), only *gnb1a* is expressed in the pineal complex (Figs. 7I–L).

### Transducin expression starts early during ontogeny

**Fig 8:**
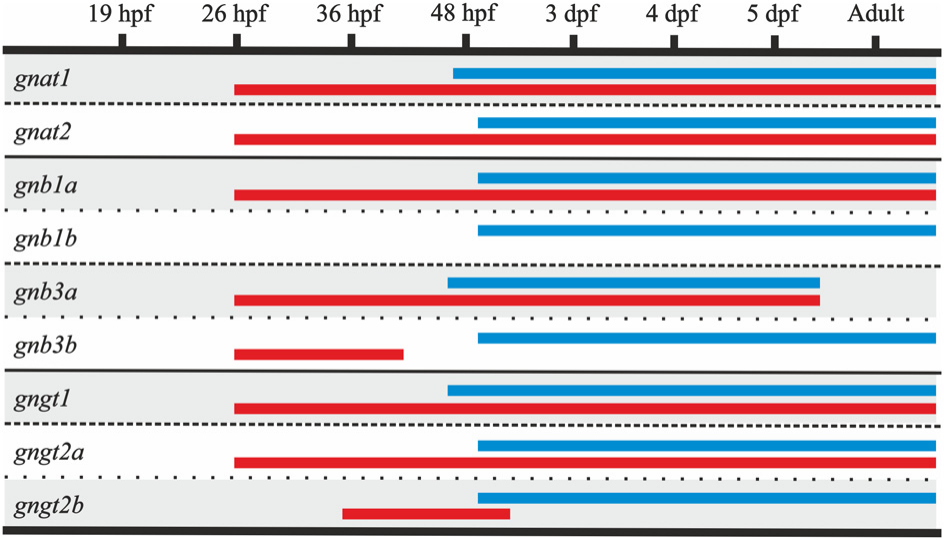
Timetable comparing the ontogeny of retina and pineal complex expression for each transducin gene. The expression in the retina (blue bars) starts either right before 48 hpf for *gnat1*, *gnb3a*, *gngt1* or slightly after 48 hpf for *gnat2*, *gnb1a*, *gnb1b*, *gnb3b*, *gngt2a* and *gngt2b*. The onset of expression in the pineal complex (red bars) is synchronised at 26 hpf for all the genes except *gnb1b*, which is not expressed at all, and *gngt2b*, which is only transiently expressed right before hatching. Furthermore, *gnb3b* also shows a transient expression, and *gnb3a* could not be detected by ISH in the adult stage in the retina or the pineal complex. Dpf; days post-fertilisation, hpf; hours post-fertilisation.

**Fig 9:**
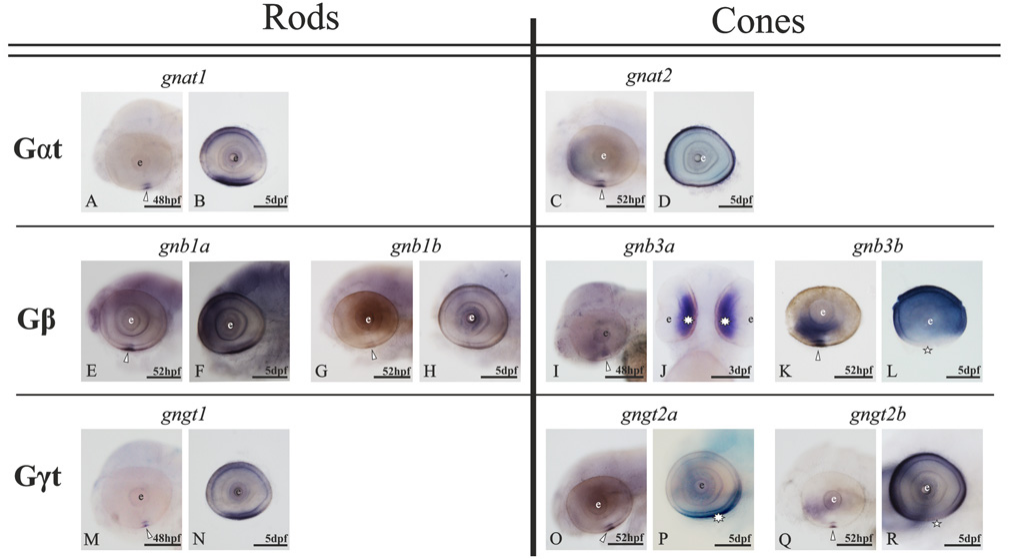
Summary of transducin gene ontogeny in the zebrafish retina. Photomicrographs of zebrafish larvae from 48 hpf to 5 dpf showing the onset and development of the expression for each transducin gene in the retina. All the pictures are lateral views with the rostral part to the left, except for panel **J** which shows a ventral view. Some eyes were dissected from the larvae for better visualisation of the staining (**B**, **D**, **K**, **L** and **N**). Expression is first observed in the ventral retina (arrowheads) for all genes, in accordance to cell differentiation: *gnat1* (**A**), *gnat2* (**C**), *gnb1a* (**E**), *gnb1b* (**G**), *gnb3a* (**I**), *gnb3b* (**K**), *gngt1* (**M**), *gngt2a* (**O**) and *gngt2b* (**Q**). For each gene, a later stage reveals the increase in the number of positive cells: panels **B**, **D**, **F**, **H**, **J**, **L**, **N**, **P** and **R**, respectively. Note the similar ontogenetic pattern for *gnb1a* (**E**, **F**) and *gnb1b* (**G**, **H**), although with weaker staining for the second, and the background-like staining along the head. In addition, note that the compartmentalisations in adults are already observable at 5 dpf: exclusively ventral expression of *gngt2a* (asterisk in **P**) as well as absence of ventral expression for the *gnb3b* (star in **L**) and *gngt2b* (star in **R**). The *gnb3a* expression can be observed in the whole retina; however, panel **J** only shows the expression at 3 dpf in the ventro-medial retina (asterisks). Scale bars: 50 μm. For abbreviations see Fig. 2 legend.

Expression of all transducin genes was analysed by WISH at 19 hours post-fertilisation (hpf), 26 hpf, 36 hpf, 48 hpf, 52 hpf, 3 dpf, 4 dpf and 5 dpf (Figs. 7, 8, 9), with the same probes used for the ISH on adult sections. At 19 hpf the retina and the pineal complex are poorly developed, therefore we used this stage as a control of non-expression but were not incorporated.

#### 1. Expression onset of the transducin genes in the retina is around 2 dpf

For all transducin genes, expression starts in the ventral retina (Fig. 9), around 48 hpf (Fig. 8), in agreement with the known cell differentiation process in the retina [36, 43]. Transcripts start to be detected at two close time points: right before 48 hpf; *gnat1* (Figs. 9A–B), *gnb3a* (Figs. 9I–J) and *gngt1* (Figs. 9M–N); and slightly after 48 hpf; *gnat2* (Figs. 9C–D), *gnb1a* (Figs. 9E–F), *gnb1b* (Figs. 9G–H), *gnb3b* (Figs. 9K–L), *gngt2a* (Figs. 9O–P) and *gngt2b* (Figs. 9Q–R). At 3 dpf, all the transducin genes are actively expressed in the retina (Fig. 8) and the photoreceptor mosaic pattern develops in the following days as previously described [21, 43]. No correlation between onset of expression and photoreceptor type was observed.

Expression was observed in the ventral retina from 52 hpf for *gnb1a* (Figs. 9E–F) and *gnb1b* (Figs. 9G–H). The only difference between the two 3R-generated *gnb1* paralogs was the intensity of the staining, which was stronger for *gnb1a* (Fig. 9F) than for *gnb1b* (Fig. 9H), like we have described in adults (Figs. 3C-D). A wide background-like staining for both genes was observed in the whole body, as previously described from 8–16-cell stage [30]. Similar wide expression was also observed for *gnb3a* (Figs. 7M–O) and *gngt2a* (Figs. 7W–Y). In all four cases, this background-like staining becomes progressively restricted to the retina and/or the pineal complex (Figs. 7, 9), while the stronger intensity of the staining in the eye is due to the higher amount of mRNA.

In contrast to adults, where no expression of *gnb3a* was detected, embryonic and larval expression of *gnb3a* was clearly observed in the retina (Figs. 9I, J) and pineal complex (Figs. 7M–O). Absence of staining in the ventral retina for *gnb3b* (Fig. 9L) and *gngt2b* (Fig. 9R) was already noticed at the 5 dpf stage, despite the cell differentiation onset occurring in this region around 48 hpf. Accordingly, exclusive expression of *gngt2a* in the ventral retina was observed at 5 dpf (Fig. 9P).

#### 2. Expression onset of the transducin genes in the pineal complex is synchronised at around 26 hpf

The ontogenetic analysis revealed synchronous onset of expression at around 26 hpf for all the transducin genes observed in the adult pineal complex (Figs. 7, 8): *gnat1* (Figs. 7A–D), *gnat2* (Figs. 7E–H), *gnb1a* (Figs. 7I–L), *gngt1* (Figs. 7S–V) and *gngt2a* (Figs. 7W–Z). In addition, *gnb3a*, whose expression was not detected in adults, was found to be expressed in the pineal complex from 26 hpf (Figs. 7M–P). As in the adult stage, no expression of *gnb1b* was detected. However, it was remarkable to find a transient expression of the cone-specific transducin paralogs *gnb3b* (Figs. 7Q, R) and *gngt2b* (Figs. 7A´, B´), from 26 to 48 hpf and 36 hpf to 3 dpf, respectively. Their expression ceases at hatching, when the transducin expression profile is completed in both the retina and the pineal complex.

Despite the background-like staining throughout the body described above for *gnb1a*, *gnb1b*, *gnb3a* and *gngt2a*, a clear but pale staining in the pineal complex could be distinguished from 26 hpf onwards for all of them, except *gnb1b*.

## Discussion

All three families of transducin subunit genes were duplicated in the early vertebrate 1R and 2R tetraploidisations [14], resulting in separate sets of subunits for rods and cones [17, 18]. Subsequently, two transducin gene families were duplicated in the teleost-specific 3R tetraploidisation; *gnb* and *gngt*, generating duplicates for rod *gnb1* and cone *gnb3* and *gngt2* [14]. Since the 3R event took place more than 300 million years ago, it can be expected that these duplicates have undergone subfunctionalisation or neofunctionalisation. The alpha subunit genes have not retained duplicates after 3R, presumably due to their central roles as primary effectors of the phototransduction cascade [14]. For those gene duplicates retained after 3R, our analyses have revealed diverse subfunctionalisations, including diverging expression patterns and expression levels. These subfunctionalisations are most likely due to differential regulation of the 3R duplicates. To identify the regulatory elements, we searched for conserved non-coding elements (CNEs) with mVISTA, using the gene nucleotide sequence plus an additional 10kb upstream sequence, for the zebrafish transducin 3R duplicates and their spotted gar orthologs. However, we did not find any significant differences. More extensive and sophisticated analyses are needed to identify any such regulatory elements.

The amino acid sequences are well conserved for each pair of duplicates in the zebrafish, with identities of 99% for the GNB1 proteins and 75% for the GNB3 and GNGT2 pairs [14]. Only three amino acid changes were found between the GNB1 duplicates, and those are not in the regions that facilitate binding to the alpha or gamma subunits. Therefore, it may be assumed that any functional differences (interactions with other components) would be minor. For the other two duplicated genes, any consequences for protein interactions will have to be investigated experimentally.

### Expression of transducin paralogs in the adult zebrafish is limited to photosensitive organs: the retina and the pineal complex

ISH experiments using transducin-specific riboprobes on adult zebrafish heads revealed positive staining limited to the primary photosensitive structures, the retina and/or the pineal complex, for all genes except *gnb3a*. In contrast, *gnb3a* expression was observed during development, suggesting temporal subfunctionalisation. However, the expression levels in 3 dpf larvae and adult eye are very similar and extremely low, which offers an explanation to the lack of staining in adults. Adult eyes have much lower levels of *gnb3a* mRNA in relation to their cell number and size. Only one expressed sequence tag for *gnb3a* (NCBI accession number: EH460287.1) was identified through BLAST searches in the NCBI EST database for zebrafish adult brain and related tissues using our probe as the query sequence. This is consistent with low expression levels in these tissues.

#### 1. Expression of transducin genes in the adult zebrafish retina is restricted to photoreceptor cells

The expression patterns of the transducin genes in the retina are conserved across vertebrate species (for references see specific cases below). We did not observe expression for any of the transducin genes in the zebrafish inner retina, in contrast to what has been found in some other vertebrates. Expression of *gnb1* and *gnb3* has been found in amacrine and bipolar cells, respectively, in different species of mammals [18, 44]. *GNB3* expression in both cones and bipolar cells appears to be highly conserved as it has also been described in frogs, chicken, mice, guinea pigs, dogs and non-human primates [20]. We have previously identified 3R duplicates of the *gnb3* gene in all investigated teleost species [14]. In goldfish, *GNB3* expression has been reported in bipolar cells [20], but the authors pointed out that there may have been some cross-reactivity. Furthermore, the goldfish has undergone a fourth tetraploidisation. Studies in other teleosts will determine if this is a common feature, and investigations in representatives from more basally radiating vertebrate lineages will be required to determine whether *GNB3* expression in bipolar cells was lost in teleosts or gained in tetrapods.

#### 2. Zebrafish gnb1 paralogs are co-expressed in rods

We found that the zebrafish *gnb1* paralogs *gnb1a* and *gnb1b* are expressed in the vast majority of rods throughout the retina, yet we cannot say with certainty that all rods express both genes. *GNB1* was initially described in rats as a rod-specific protein [18], and subsequently identified in many different vertebrate species. Although the retention of two almost identical zebrafish *gnb1* paralogs suggests specialisation by diverging morphological or temporal expression, we did not observe such differences. The continuous expression of *gnb1b* and the rhythmic oscillation of *gnb1a* expression suggest a dosage effect, or that one of the genes is expressed in an organ we have not investigated, probably the one with the lowest expression, *gnb1b*. Higher expression of *gnb1a* at ZT8 could be related to the higher visual sensitivity demonstrated for zebrafish in the afternoon [45]. However, no clear explanation was found for a peak of expression at ZT16.

The co-expression of this pair of paralogs is peculiar, but not unique within the phototransduction cascade. We have recently found a similar case between the *pde6ga* and *pde6gb* paralogs (work in progress) and it was also suggested for the arrestin genes, where both *arrSa* and *arrSb* paralogs are expressed (and very likely co-expressed) in rods [46] and the opsin GPCR kinases *grk1a* and *grk1b* [47]. We postulate that there might be two parallel phototransduction pathways within rods, at least for a few steps of the pathway.

#### 3. Cone transducin paralogs retained after 3R show compartmentalisation

Two cone-specific transducin paralog pairs have been retained after 3R: *gnb3a/gnb3b* and *gngt2a*/*gngt2b*. We found that both pairs display compartmentalisation, but in quite different ways. The *gngt2* paralogs display a beautiful case of spatial subfunctionalisation, where *gngt2a* is expressed exclusively in the ventral retina and *gngt2b* is expressed in the dorsal and medial retina (Fig. 6). For the *gnb3* paralogs, *gnb3b* is expressed in the dorsal and medial retina, while very little expression of *gnb3a* was revealed by RT-qPCR in adult eye, which was undetectable by ISH. Consequently, the functionality of the cones in the ventral retina could be questioned since they seem to have none or very little beta transducin subunit. However, a recent publication demonstrated that *gnb3* knock-out mice produce stable responses with normal kinetics and saturating amplitudes, although with an approximately fourfold reduction in sensitivity [48]. We propose that a reduced sensitivity of the ventral retina may have evolved as a protective mechanism. Zebrafish live in shallow water, in subequatorial rivers, so the direct light incidence from above is high and could be harmful. The ventral retina has been shown to have a higher number of photoreceptors, as well as shorter photoreceptor outer segments and thicker RPE [40], specialisations that may also be protective. Furthermore, light-induced photoreceptor degeneration experiments always damage the dorsal retina more severely [49], suggesting a reduced sensitivity of the ventral part.

**Fig 10:**
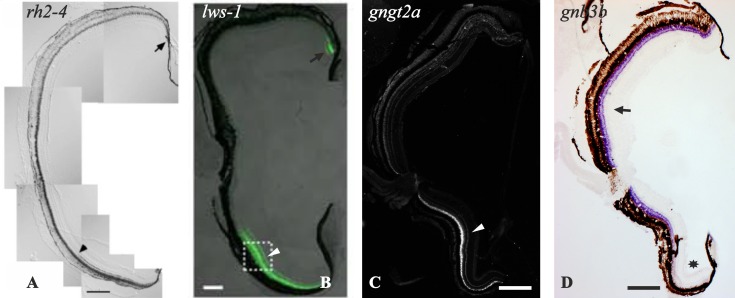
Photomontage that highlights gene specialisations of the zebrafish ventral retina. These photomicrographs show radial sections of adult zebrafish retinae. They demonstrate specific expression of *rh2–4* (**A**), *lws-1* (**B**) and *gngt2a* (**C**) in the ventral retina (arrowheads), while *gnb3b* (**D**) shows expression in the dorsal and medial retina (black arrow). Note that *gnb3b* expression is absent in the ventral retina (asterisk). The pictures in panels **A** and **B** are used with permission from Takechi and Kawamura (2005) [51] and Tsujimura *et al*. (2010) [50], respectively. The pictures in panels **C** and **D** are extracted from the present results. Scale bars: 200 μm.

These observations provide evidence not only for morphological but also molecular specialisations of the ventral retina. In addition to the *gnb3* paralogs, other phototransduction genes have been found to have specialised paralogs that are exclusively expressed in the ventral retina: the red opsin *lws-1* [50], the green opsin *rh2–4* [51], and the *gngt2a* (present results, see Fig. 10).

Aside from opsins and transducins, there are other gene families involved in zebrafish vision that show subfunctionalisations after 3R or due to species/lineage-specific (local) duplications: guanylyl cyclase [52], guanylyl cyclase activating protein [53, 54], arrestin [46], G protein-coupled receptor kinase [47, 55] and retinoid binding protein [56]. However, none of these show compartmentalisation in the ventral retina, or have not yet been investigated in this regard.

### Impact of 3R on transducin genes in the pineal complex: partial compartmentalisation of *gnb1* paralogs

Comparative studies of the pineal complex are complicated due to its variability across species. In zebrafish, the pineal organ contains two types of light-sensitive photoreceptor cells that share extensive similarities with retinal rods and cones, such as cell morphology, responses to light stimuli, and expression of components involved in phototransduction [8, 55, 57–61].

**Fig 11:**
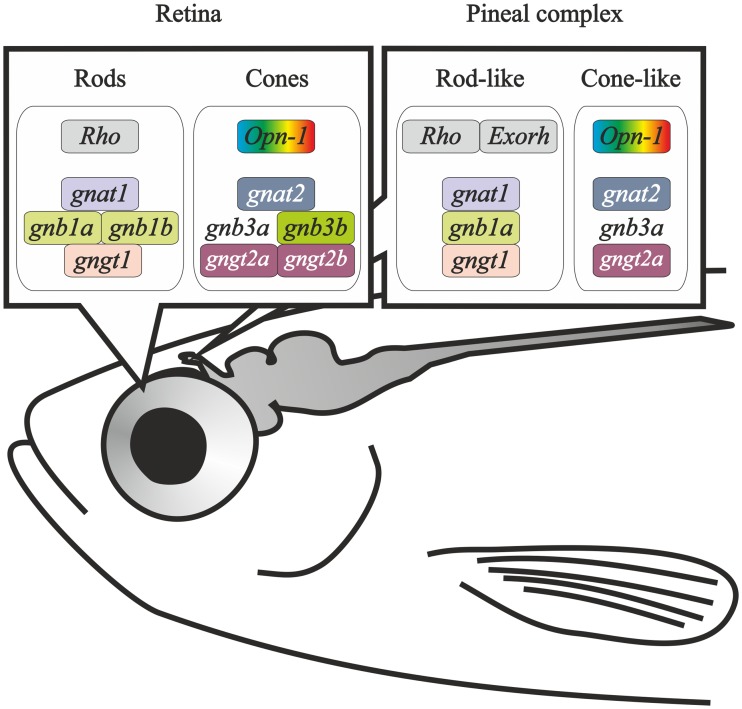
Opsin-transducin model suggested for the retina and pineal complex photoreceptors. Drawing of an adult zebrafish head showing the opsin and transducin genes expressed in rods and cones of the retina, as well as a suggested model for the rod- and cone-like photoreceptors of the pineal complex. Due to the low expression of *gnb3a* in the adult retina, and the fact that its expression could not be demonstrated in the adult pineal complex, it is shown without a box.

In adults, only *gnat2* expression has been reported [61]. In addition to this, we describe here the expression of *gnat1*, *gnat2*, *gnb1a*, *gngt1* and *gngt2a*, but not *gnb1b*, *gnb3a*, *gnb3b* and *gngt2b* in the pineal complex. The identified subunits would be able to form a rod-like transducin heterotrimer (*gnat1*-*gnb1a*-*gngt1*; Fig. 11), while only alpha and gamma subunits for the cone-like transducin are present (*gnat2*-*gngt2a*; Fig. 11). The lack of detectable *gnb3a* in the adult pineal complex could be due to temporal subfunctionalisation or to very low expression levels, like in the retina, as its expression was found in both organs early in development. Nevertheless, studies analysing the amount of GNB3a protein and its co-localisation with opsins must be carried out in order to investigate the functionality of a possible cone-like transducin.

The lack of expression of *gnb1b* in the pineal complex suggests a partial compartmentalisation for the *gnb1* paralogs: *gnb1a* is expressed in both the retina and the pineal complex, while *gnb1b* is restricted to the retina. No other case of partial compartmentalisation has been reported for the zebrafish phototransduction proteins.

It is not clear whether the pineal complex represents a primitive stage during eye evolution, or if it has evolved largely independently from the vertebrate eye [7]. In contrast to the retina, the structure of the pineal organ has changed dramatically in mammals relative to non-mammalian vertebrates as a result of the progressive replacement of direct to indirect photosensitivity [41]. The presence of the components needed to form functional rod- and cone-specific phototransduction pathways has been demonstrated previously in pineal cells of neonatal rats, although expression of many of these genes decline rapidly during development. These findings strongly support loss of photosensitivity in the mammalian pineal organ during ontogeny [62]. In contrast, lampreys [11], zebrafish ([26], present results), other teleost fishes [9], as well as amphibians and reptiles (see [10, 41]) seem to maintain functionality for the two photoreceptor cell types during their entire lifespan. Thus, a complete analysis of all the phototransduction proteins expressed in the zebrafish pineal organ and their cell-type specificity would offer important insights into this interesting possibility.

### The ontogenetic analysis of the transducin genes reveals early establishment of the adult pattern and two partial subfunctionalisations

This comparative ontogenetic analysis of the transducin genes between the zebrafish retina and pineal complex shows an earlier onset of the expression in the pineal complex than in the retina. Similar results have previously been reported for the GNAT [27, 59] and GNGT subunits [26, 32]. Earlier expression in the pineal organ compared to the retina has been also reported for opsins in lampreys [63] and teleost fishes [64–66]. These results are not surprising, as the two organs have different embryonic origins and their maturation is differentially regulated [67]. The early onset of genes involved in phototransduction in the pineal has been suggested previously to relate to regulation of hatching [66].

#### 1. The early establishment of the adult pattern highlights the importance of the ventral retina in the larvae

Retinal differentiation in zebrafish starts in the ventral region, different from most other vertebrates, in which it is initiated in central locations [21]. The sequential events leading to photoreceptor differentiation can be summarised as follows: rhodopsin expression starts at 50 hpf, colour opsins at 52 hpf [43], outer segment development starts at 54 hpf, synaptic ribbons are discernible at 62 hpf and around 74 hpf the eyes respond to light [21].

A general overview of the ontogeny of the transducin genes reveals onset of expression in the retina around 48 hpf (Fig. 8), which is similar to the opsins [51] and PDE6 (unpublished results). All three cases are in accordance with the development of the visual system, but are not consistent with the idea that rods do not become functional until around 10 dpf [68, 69]. A difference between rods and cones in onset of functionality has been demonstrated previously and attributed to different retinoic acid levels [21]. However, to our knowledge, the reason for this long delay in acquiring functionality in putative “fully-developed” rods is not yet known.

In addition, ontogenetic analyses of the transducin genes show early establishment of the adult expression patterns, i.e., the exclusive ventral expression of *gngt2a* and absence of a cone beta subunit in the ventral retina. This suggests an important role of the ventral retina from an early point in development, a pattern that has been demonstrated previously for the opsins [50, 51]. Taken together, this supports the protective hypothesis of the ventral retina since these specialisations would provide exclusive expression of a few genes related to light detection in the newly hatched larvae, during a time when they predominately swim at the bottom of a stream or tank with light incidence mainly from above [21].

Developmental analyses, including the present study, have revealed ubiquitous expression of *gnb1a*, *gnb1b*, *gnb3a* and *gngt2a* in the whole body from very early in development ([70], present results), which gradually becomes restricted to the retina and/or the pineal complex during the larval stage ([71, 30], present results). Detailed ontogenetic analysis of the GNB subunits have revealed a similar spatiotemporal plan for both *gnb1a* and *gnb1b*: both mRNAs are first observed at the 8–16 cell stage [30] and both genes are involved in the migration and polarisation, but not differentiation, of the primordial germ cells [30]. These data, together with the testis expression of *gngt1* [26], suggest a crucial role of the “rod-specific” βγ dimer in the development of the gonads, a process that is not complete until around 30 dpf [72]. This coincides with the time-point when the adult eye has reached its mature size [68]. By this time-point, *gnb1a*, *gnb1b* and *gngt1* are completely restricted to the photosensitive structures in the head. In the retina, *gnb1a* and *gnb1b* follow an identical developmental pattern.

#### 2. Two partial subfunctionalisations between the retina and the pineal complex from the larval stage

The ontogenetic analysis of the pineal complex reveals expression of *gnat1*, *gnat2*, *gnb1a*, *gnb3a*, *gngt1* and *gngt2a* from 26 hpf. This would facilitate the formation of functional rod-like (*gnat1*-*gnb1a*-*gngt1*; Fig. 11) and cone-like transducin heterotrimers (*gnat2*-*gnb3a*-*gngt2a*; Fig. 11). This is different from the adult pineal complex where expression of *gnb3a* could not be demonstrated. Moreover, we found an interesting transient expression of *gnb3b* and *gngt2b* in the pineal complex prior to hatching. This finding reveals a partial temporal subfunctionalisation, as both genes are expressed in the retina from 48 hpf. Their functional consequences are not clear, but the timing implies a role in hatching regulation [66]. Partial compartmentalisation was also observed between the retina and the pineal complex for *gnb1a* and *gnb1b*.

## Conclusions

The evolutionary consequences of 3R for the zebrafish transducin subunits are manifold. Three paralogous pairs have been retained after 3R: *gnb1a*/*gnb1b*, *gnb3a*/*gnb3b* and *gngt2a*/*gngt2b*, showing a very interesting range of specialisations. *Gnb1a* and *gnb1b* genes are co-expressed in the same rods and *gnb1b* is absent from the pineal complex, which indicates a partial compartmentalisation. The cone-specific *gnb3b* gene is expressed in the dorsal and medial retina, while the expression of *gnb3a* in adults is so low that it cannot be detected by ISH. Similar expression levels of *gnb3a* were found in 3 dpf larvae, where ISH staining can be observed in both the retina and the pineal complex. Additionally, *gnb3b* mRNA is only transiently detected in the pineal complex prior to hatching, indicating a partial temporal subfunctionalisation. The cone-specific *gngt2b* gene is expressed in the dorsal and medial retina, while *gngt2a* is exclusively expressed in the ventral retina. This is a very clear case of compartmentalisation that highlights an important specialisation of the ventral retina. In addition, *gngt2b* has undergone a process of partial temporal subfunctionalisation similar to that of *gnb3b*. Taken together, these results show multiple examples of altered gene expression reflecting anatomical and developmental specialisations of 3R paralogs.
